# Safety and Efficacy of Epithelium-On Corneal Collagen Cross-Linking Using a Multifactorial Approach to Achieve Proper Stromal Riboflavin Saturation

**DOI:** 10.1155/2012/498435

**Published:** 2012-07-30

**Authors:** Aleksandar Stojanovic, Xiangjun Chen, Nan Jin, Ting Zhang, Filip Stojanovic, Sten Raeder, Tor Paaske Utheim

**Affiliations:** ^1^Department of Ophthalmology, University Hospital of North Norway, Fløyvn. 32, Tromsdalen, 9020 Tromsø, Norway; ^2^SynsLaser Kirurgi, 9008 Tromsø and 0159 Oslo, Norway; ^3^School of Ophthalmology and Optometry and Eye Hospital, Wenzhou Medical College, Zhejiang, Wenzhou 325003, China; ^4^University of Tromsø, 9037 Tromsø, Norway; ^5^Department of Ophthalmology, Stavanger University Hospital, 4011 Stavanger, Norway; ^6^Department of Clinical Medicine, University of Bergen, 5020 Bergen, Norway; ^7^Department of Medical Biochemistry, Oslo University Hospital, 0424 Oslo, Norway

## Abstract

*Purpose*. To evaluate the efficacy and safety of epithelium-on corneal collagen cross-linking (CXL) using a multifactorial approach to achieve proper stromal riboflavin saturation. *Methods*. This non-randomized retrospective study comprised 61 eyes with progressive keratoconus treated with epithelium-on CXL. Chemical epithelial penetration enhancement (benzalkonium chloride-containing local medication and hypotonic riboflavin solution), mechanical disruption of the superficial epithelium, and prolongation of the riboflavin-induction time until verification of stromal saturation were used before the UVA irradiation. Uncorrected and corrected distance visual acuity (UDVA, CDVA), refraction, corneal topography, and aberrometry were evaluated at baseline and at 1, 3, 6, and 12 months postoperative. *Results*. At 12-month, UDVA and CDVA improved significantly. None of the eyes lost lines of CDVA, while 27.4% of the eyes gained 2 or more lines. Mean spherical equivalent decreased by 0.74 D, and mean cylindrical reduction was 1.15 D. Irregularity index and asymmetry from Scheimpflug-based topography and Max-*K* at the location of cone from Placido-based topography showed a significant decrease. Higher-order-aberration data demonstrated a slight reduction in odd-order aberrations *S* 3, 5,7 (*P* = 0.04). Postoperative pain without other complications was recorded. *Conclusion*. Epithelium-on CXL with our novel protocol appeared to be safe and effective in the treatment of progressive keratoconus.

## 1. Introduction

Corneal collagen cross-linking (CXL) is a low-invasive treatment aimed to improve biomechanical stability in eyes with keratectasia [1–3]. A photodynamic reaction induced by photosensitizing riboflavin and ultraviolet A (UVA) light causes an increase of the number of intra- and interfibrillar covalent bonds and the corneal collagen resistance against enzymatic degradation [4–6]. Increased stromal biomechanical strength and lamellar compaction lead to stabilized corneal shape and better corneal symmetry, potentially causing an improvement in visual function [2, 7–9]. 

In CXL, riboflavin has a dual function acting both as a photosensitizer inducing the physical collagen cross-linking and as an absorber of the UVA irradiation, preventing damage to deeper ocular structures [10, 11]. Proper corneal stromal saturation with riboflavin is therefore essential in CXL, and without its presence the UVA radiation may cause the collagen fibers to degrade rather than to facilitate cross-linking [12].

 The “standard CXL protocol” described by Wollensak and colleagues includes removal of the corneal epithelium in a diameter of 9 mm, followed by saturation of the corneal stroma using 0.1% isotonic riboflavin solution in 20% dextran [13]. This procedure is proved to be effective in increasing corneal stiffness [13], stabilization of keratoconus, and in some cases in improving the refractive and topographic features [14, 15]. Even so, the epithelial removal may lead to serious complications that include infection [16, 17], stromal haze [18], and corneal melting [19] in addition to severe pain and decrease in vision occurring during the first days after the treatment. To avoid such complications, Boxer Wachler et al. suggested a modification of the technique by keeping the epithelium intact (epithelium-on or transepithelial CXL) [20]. However, finding appropriate means of increasing corneal epithelial permeability prior to riboflavin application was warranted as riboflavin has a molecular weight of 338 Da, whereas the corneal epithelium is impermeable to compounds with a molecular weight greater than 100 Da [21]. Accordingly, various approaches have been tried clinically and in the laboratory to enhance the epithelial permeability before the riboflavin application. Chemical enhancers such as benzalkonium chloride (BAC), ethylenediaminetetraacetic acid (EDTA), gentamycin, tetracaine, and 20% ethanol [22–25] were used, as well as partial grid-like pattern deepithelialization [22], excimer laser superficial epithelial removal [26], and the replacement of the isotonic by hypotonic riboflavin solution [24, 27]. The results varied between the studies, but the majority of the aforementioned methods lead to increased epithelial permeability for riboflavin. 

 In the current study, a multifactorial approach was utilized to enhance the riboflavin penetration by employing: (1) BAC-containing local medication; (2) hypotonic riboflavin solution without dextran; (3) increased riboflavin solution concentration; (4) mechanical disruption of the superficial epithelium (microabrasions); (5) prolongation of the riboflavin-induction time until objective verification of the stromal saturation is confirmed. By such an approach, this nonrandomized retrospective study aimed to evaluate the efficacy and safety of the epithelium-on CXL in treatment of progressive keratoconus. 

## 2. Patients and Methods

In this retrospective, interventional case series, we reviewed the medical records of all patients with advanced progressive keratoconus who had 12-month observation time after the epithelium-on CXL treatment using our multifactorial approach. The treatment was performed at The Eye Department of the University Hospital North Norway, Tromsø, Norway, between September 15, 2009 and September 15, 2010. This study was approved by the regional ethics committee and adhered to the official ethical regulations for clinical research and the Tenets of the Declaration of Helsinki. Inclusion criteria included (1) documented progression of keratoconus during the last 12 months before treatment (increase of astigmatism or myopia by 1.00 D or increase in average SimK by 1.50 D); (2) minimum corneal thickness of no less than 400 *μ*m at the thinnest point measured by ultrasound pachymetry; (3) age ranging from 18 to 45 years; (4) Amsler-Krumeich keratoconus classification stage II to III. Exclusion criteria were: (1) history of herpes virus keratitis; (2) severe dry eye; (3) concurrent corneal infections; (4) previous ocular surgery; (5) hard contact lens wear ≤4 weeks before the baseline examination.

Pre- and postoperative assessments consisted of slit lamp biomicroscopy, Scheimpflug-based corneal topo-/tomography (Precisio, iVIS Technology, Taranto, Italy), Placido disk-based topography and wavefront aberrometry (OPD-Scan II, Nidek. Co., Ltd. Aichi, Japan), uncorrected (UDVA) and corrected (CDVA) distance visual acuities (Nidek RT 2100 system, Nidek Co. Ltd., Aichi, Japan), ultrasound pachymetry (Cornea-Gage Plus, Sonogage Inc., Cleveland, Ohio), tonometry (Icare tonometer, Revenio Group Corporation, Helsinki, Finland), and patients′ subjective evaluation of postoperative pain. The patients were examined at 1, 3, 6, and 12 months postoperative.

### 2.1. Surgical Technique

**Figure 1:**
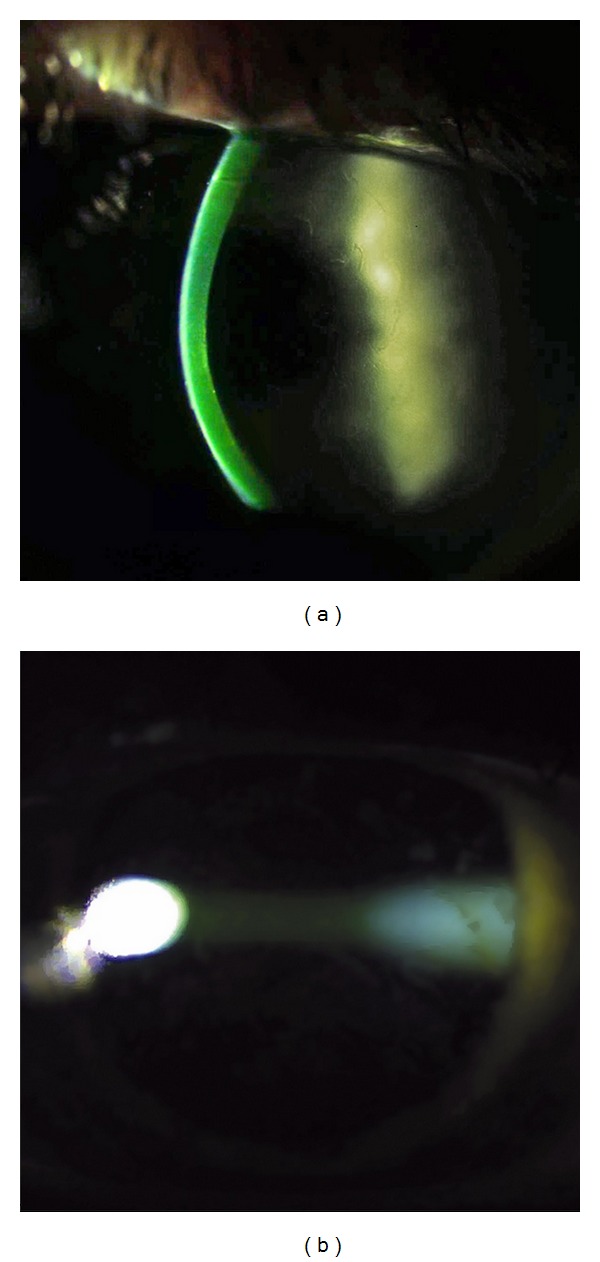
. Slit lamp verification of the stromal riboflavin saturation before the UVA irradiation.

To reduce the risk for UV exposure of retroiridal eye structures, miosis was induced by applying two drops of pilocarpine 2% (Pilokarpin, Ophtha AS, Norway). It was followed by the application of two drops of local anesthetic proparacaine 0.5% (Alcaine, Alcon Norway AS), two drops of local antibiotic gentamycin 0.3% (Garamycin, Schering-Plough AS, Norway) followed by proparacaine again, one drop every minute for five minutes. All the drops were preserved by BAC (0.001% for Pilokarpin, 0.005% for Garamycin and 0.01% for Alcaine), aiming to increase the epithelial permeability by chemically disrupting the tight junction proteins. A round Merocel sponge (Medtronic, Inc., Minneapolis, MN) of 5 mm in diameter was inserted into the conjunctival sac to provide a depot of riboflavin, and to produce microabrasions of the superficial epithelial layers caused by friction upon patient's blinking. Thereafter, two drops of proparacaine and two drops of 0.5% aqueous riboflavin solution *without dextran* (Vitamin B2; Streuli, Uznach, Switzerland) were applied alternating every 30 seconds, until the riboflavin saturation was verified by the slit-lamp inspection of the cornea and by the determination of the presence of riboflavin flare in the anterior chamber (Figure 1). Under the same examination the staining of the epithelial microabrasions was verified. The initial slit-lamp saturation evaluation was performed 25 minutes after the first application of riboflavin and repeated every five minutes until the saturation was confirmed. During the premedication and riboflavin induction time the patient was instructed to blink normally between eye drop instillation and to remain in a comfortable sitting position. The Merocel sponge was then removed and corneal thickness measured with ultrasound pachymetry, at which point the patient was placed in supine position. Irrigation with isotonic balanced salt solution (BSS) was performed before the UVA irradiation in order to avoid the shielding effect of riboflavin covering the epithelium. An eyelid speculum was then inserted, and a ring-shaped Merocel shield k20-5021 (Katena Products, Inc. Denville, NJ) was applied to protect the limbal region and its stem cells from UVA radiation.

The cornea was subjected to UVA radiation for 30 minutes with a wavelength of 365 nm at a working distance of 5 cm. The UV-X lamp (IROC AG, Zürich, Switzerland) provided an irradiance of 3 mW/cm^2^ within a circular diameter of 9 mm. During the irradiation, BSS was applied every three minutes, and proparacaine drops were added as needed.

After the UVA irradiation, two drops of atropine 1% (Atropin minims, Chauvin, England) and 2 drops of gentamycin were applied. The cornea was protected with a soft bandage contact lens for 12–18 hours. Instructions were given to apply a mixture of 0.1% dexamethasone and 0.5% chloromycetin (Spersadex med Kloramfenikol, Novartis, Norway) eye drops four times daily for seven days, as well as to use artificial tears as needed.

### 2.2. Statistical Analysis

All visual acuity values were recorded as Snellen values, converted to LogMAR for statistical analyses and then changed back to Snellen values for presentation purposes. Pre- and postoperative topography was analyzed using Precisio's irregularity index (IRI) as well as by measuring the central 5 mm using OPD indices. Statistical analysis was performed to compare the post-operative data with the preoperative data using the paired *t*-test with IBM SPSS Statistics v19.0 (IBM, Armonk, NY). *P* < 0.05 was considered statistically significant.

## 3. Results

Sixty-one eyes of 53 patients fulfilled the inclusion and exclusion criteria. The mean age of the patients was 32 ± 10 years (range, 15–52 years). 85% of the eyes (52 eyes) were from male patients.

### 3.1. Visual Acuity

**Figure 2:**
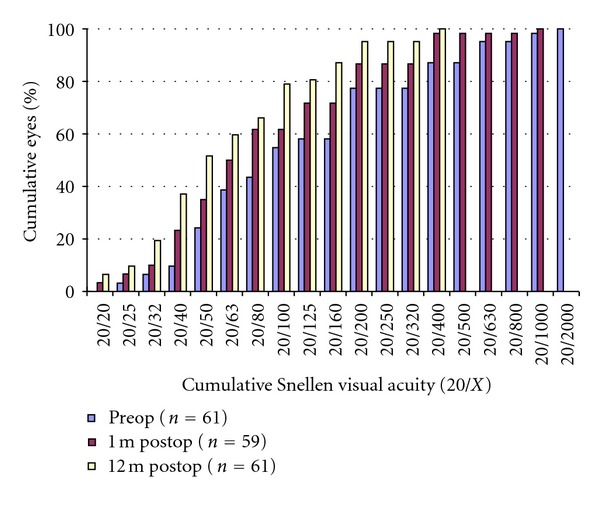
UDVA 1 months and 12-months after the epithelium-on CXL.

**Figure 3:**
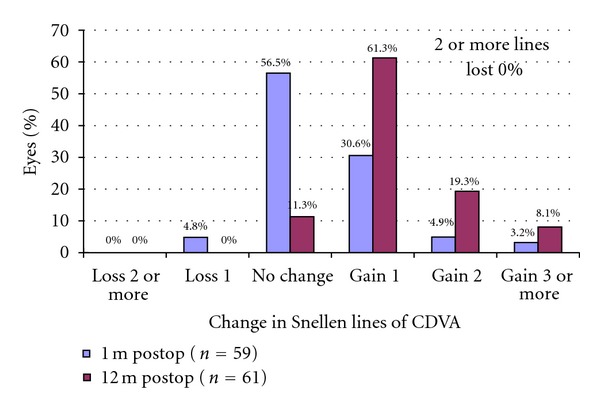
Gain/loss of CDVA 1 months and 12 months after epithelium-on CXL.

**Figure 4:**
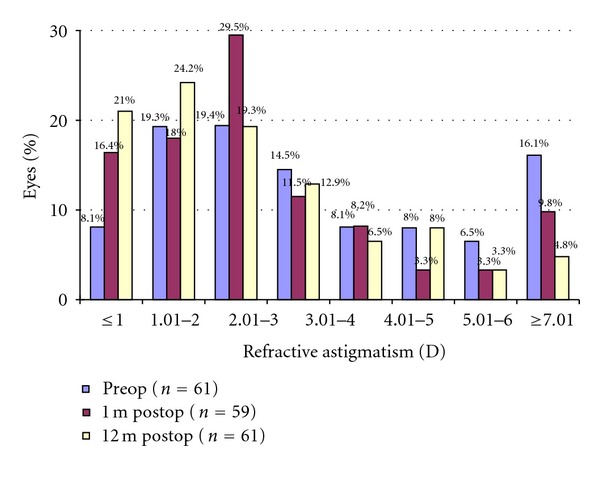
Refractive astigmatism after epithelium-on CXL.

**Figure 5:**
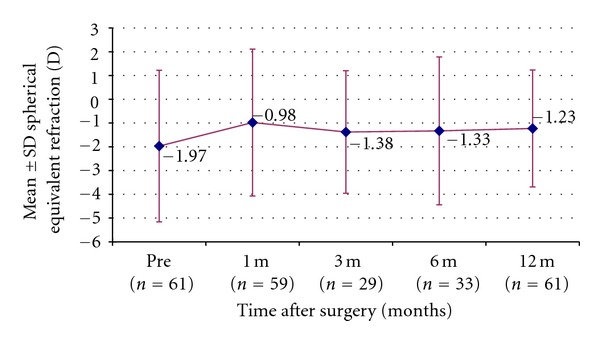
Stability of SE after epithelium-on CXL.

Figures 2, 3, 4, and 5 and Table 1 show the visual acuity and refraction measurements pre- and postoperatively. The UDVA and CDVA improved significantly (*P* < 0.05). At 12-month followup, none of the eyes lost lines of CDVA, while 27.4% of the eyes gained 2 or more lines and the safety index was 1.14. At the same time point, mean spherical equivalent refraction decreased by 0.74 D (less myopic, *P* = 0.05), while mean cylinder decreased by 1.15 D (*P* = 0.00).

### 3.2. Corneal Topography and Wavefront Aberrometry

**Figure 6:**
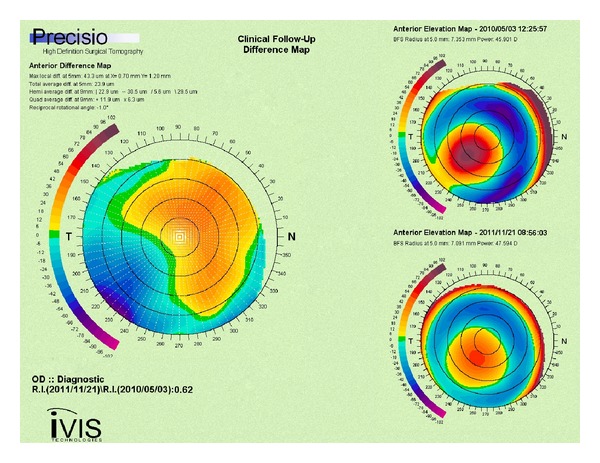
Scheimpflug anterior elevation difference map showing depression at the cone and increased elevation orthogonally (left image). Scheimpflug anterior elevation maps (right images): preoperative (upper) and 12-months after epithelium-on CXL (lower).

Table 2 shows the postoperative changes of topography and aberrometry. Data from the Precisio showed reduction in posterior elevation (*P* = 0.01), irregularity index (*P* = 0.01), and asymmetry (*P* = 0.01). The *K*-value from OPD did not significantly alter regarding Mean-*K*, while the Max-*K* showed a significant decrease (*P* = 0.02) at the location of the cone.  Figure 6 shows an example of the topographic changes in one of the treated eyes. 

Aberrometry data showed a reduction in odd-order- S 3,5,7 (*P* = 0.04) and total higher-order-aberrations (*P* = 0.05). 

### 3.3. Pachymetry

Precisio-measured pachymetry in Table 1 shows decrease in thickness at 1-month followup (*P* = 0.00) and thereafter a gradual increase to preoperative level at 12 months after the treatment (*P* = 0.15). 

### 3.4. Pain Evaluation

Patients reported moderate to severe postoperative pain during the first 4–12 hours, peeking at 4–6 hours after surgery.

### 3.5. Complications

Discreet superficial epithelial layer damage could be observed on slit-lamp examination upon the verification of riboflavin saturation and after the CXL treatment. No serious complications were recorded during the follow-up period.

## 4. Discussion

Previous studies report conflicting results on the effects of epithelium-on CXL. While Pinelli and colleagues reported no significant difference in the analyzed parameters between epithelium-on and standard CXL [28], Wollensak and Iomdina found that the corneal biomechanical stiffening after epithelium-on CXL was about one-fifth compared to the epithelium-off CXL in an animal model [4]. Other clinical and laboratory studies have reported weaker or no effect of CXL using the epithelium-on method [22, 23, 25, 29–31]. Collectively, the studies suggested that the significantly weaker biomechanical effect of epithelium-on CXL was due to the insufficient and inhomogeneous transepithelial riboflavin diffusion into the corneal stroma. However, a limitation of most of the studies that procured the low cross-linking effect or low stromal saturation of riboflavin with the epithelium-on CXL includes the use of the standard—or only slightly modified—Wollensak/Seiler protocol on nondeepithelialized eyes. Moreover, the authors did not attempt to enhance the riboflavin penetration, effectively only showing that the epithelium-on CXL does not work with the standard epithelium-off protocol. Intriguingly, epithelial permeability can be enhanced by application of several tensioactive substances including BAC and gentamicin at concentrations normally used in industrial preparations [32]. Such pharmacological enhancements, which are commonly used in epithelium-on CXL [20, 33], were included in the current protocol.

On the basis of previous studies reporting increased epithelial riboflavin permeability using hypotonic solution compared to isotonic solution [24, 27, 34], hypotonic solution was applied in the current protocol. Furthermore, we avoided the use of riboflavin solution with dextran due to its high viscosity, which inhibits the penetration through the epithelium [35]. Hypotonic riboflavin was originally used with epithelium-off CXL protocol to induce significant edema in corneas thinner than 400 *μ*m [27]. However, the swelling of the corneas with intact epithelium seems to be of a considerably lower degree. In the present study, only around 10 microns of swelling were recorded in a subgroup of 29 eyes, presenting respective mean pachymetry of 450.50 ± 42.90 and 471.65 ± 41.43 *μ*m before and after the corneal saturation with the 0.5% hypotonic riboflavin solution. Even though the clinical safety of CXL with hypotonic riboflavin solution has been documented [36], issues of the corneal endothelial cell toxicity were considered because of the decreased UV-protective effect with hypotonic riboflavin due to the decrease of UV absorption coefficient from *≈*53 cm^−1^ for 0.1% *isotonic* Riboflavin solution to *≈*42 cm^−1^ for 0.1% *hypotonic* riboflavin solution [36]. To compensate for this, increasing the concentration of riboflavin in the hypoosmolar solution may enhance UVA absorption [37] and hence decrease the UV-radiation at the endothelial level. The current study addresses the endothelial safety as hypotonic riboflavin concentration of 0.5% was applied. A secondary benefit of the increased concentration is the presumably increased availability of the riboflavin molecules to penetrate the epithelium and saturate the stroma. In a subgroup analysis of 21 eyes performing pre- and postoperative specular microscopy using Konan CellCheck XL (Konan Medical, Irvine, CA), the endothelial count decreased insignificantly (*P* = 0.09) from 2738 ± 188 cells/mm^2^ to 2608 ± 311 cells/mm^2^.

The current protocol also employed mechanical scarification of the epithelial surface by creation of microabrasions caused by movement of a Merocel sponge over the corneal surface with patient's blinking. The amount of such micro-abrasions could obviously not be standardized, and it varied between cases. This may be the reason for a relatively large variation in saturation time (mentioned in the next paragraph).

Finally, in addition to the chemical and mechanical enhancements, the current protocol demanded a slit-lamp verification of the stromal saturation before the UVA radiation (Figure 1). Our clinical observations showed that riboflavin saturation was achieved after 25–45 minutes, so that a commonly used set induction time of, for example, 30 minutes would in many cases lead to insufficient riboflavin concentration in the stroma.

Our visual and refractive outcomes were comparable to other published CXL studies. In the current study there were no cases with a loss of ≥2 lines of CDVA, endothelial cell count did not change significantly, and there were no infections or other types of keratitis. 

Most of the patients reported pain peaking 4–6 hours after the treatment despite the mostly preserved epithelium. This may be explained by actinic-keratoconjunctivitis-like reaction caused by the UV exposure during the treatment and by the microabrasions caused on purpose, to enhance riboflavin penetration. 

Corneal topography change in curvature has often been used to evaluate the effect of CXL [23, 38, 39]. In our study, the maximum-*K* value and DSI (differential sector index) decreased significantly on the OPD II, Placido-based topography, as did the posterior elevation, irregularity index and asymmetry on the Precisio, Scheimpflug-based topography. However, our mean-*K* did not decrease in contrast to most other studies. In most of our cases, in addition to the Max-*K* decrease, a moderate increase in steepness on the opposite side of the cone occurred (Figure 6), resulting in only minor decrease of the mean-*K* but more symmetrical corneal optics, decreased higher-order-aberrations, and improved vision. We hypothesize that this may be a consequence of a possibly heavier riboflavin load in the inferior corneal stroma due to the sitting position and blinking during the riboflavin induction, leading to a locally increased cross-linking effect. This theory warrants a further study and may be a small step in the direction of “customized” CXL.

**Figure 7:**
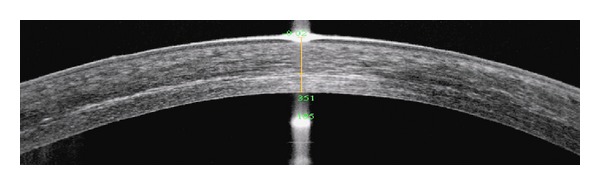
AS-OCT image showing demarcation line at 351 *μ*m, 2 months after epithelium-on CXL.

Detection of the demarcation line [40] after CXL has been considered the proof of the efficacy and the measure of the depth of the corneal cross-linking. Although the precise nature and significance of the demarcation line (increased optical density) in relation to the cross-linking process are uncertain, it may be consequent to the keratocyte apoptosis and their subsequent repopulation [41]. Keratocyte apoptosis has to a lesser extent been demonstrated after epithelium-on CXL [4]. Filippello et al. epithelium-on CXL with 0.1% isotonic riboflavin solution showed that the demarcation line two weeks postoperatively was located approximately 100 *μ*m from the corneal epithelium [23]. In 24 eyes treated with the current protocol that could be evaluated by RTVue (Optovue Inc., Fremont, CA) AS-OCT (anterior segment optical coherence tomography), the mean demarcation line was located at the depth of 316.92 ± 49.16  *μ*m (range 260 to 367) from the surface (Figure 7), which is close to the observations after epithelium-off CXL. 

Epithelial absorption/filtering of the UVA light that could potentially lead to lesser energy delivered to riboflavin-saturated stroma has also been stated as an argument against the use of epithelium-on CXL. Different studies offer a variety of evaluation backgrounds of this matter. A study performed by Baiocci et al. [21] claimed that human corneal epithelium and the underlying basement membrane naturally absorb 30% to 33% of UVA radiation (400 to 350 nm), while other studies showed that the epithelial UV absorption occurs only with wavelengths lower than 310 nm [42–44]. We may assume that the UV-absorption of the riboflavin within the epithelium is probably low (since the epithelial cells are hydrophobic and do not absorb riboflavin) and that the epithelial interstitial space is of negligible volume. The current protocol includes washing off the riboflavin from the corneal surface before the UVA-radiation in order to minimize the UV-energy loss due to its possible absorption by the riboflavin.

Finally, even if the epithelium-on CXL leads to a shallower cross-linking compared to the epithelium-off, the density of collagen fibers in corneal stroma is much higher in the anterior portion where most of the collagen cross-links occur [40, 45]. 

## 5. Conclusion

This retrospective study showed that epithelium-on CXL using our novel protocol appeared to be effective and safe in treating progressive keratoconus. The improvements in the visual, refractive, and topographic parameters in our patients indicate that epithelium-on CXL had sufficient effect to halt the progression of keratoconus and improve the corneal shape. A randomized controlled trial is warranted to verify that the effect of the current approach is comparable with the standard epithelium-off CXL. Furthermore, the combination of different enhancers, osmolality, and concentration of riboflavin should be explored to further improve the procedure.

## Figures and Tables

**Table 1:** Visual acuity, refraction, and corneal thickness changes during 1-year followup.

Parameter	Preoperative (*n* = 61)	Postoperative			
		1 Month (*n* = 59)	3 Months (*n* = 29)	6 Months (*n* = 33)	12 Months (*n* = 61)
VA (Snellen)					
UDVA	(20/133 ± 20/57)	(20/95 ± 20/49) (*P* = 0.00)	(20/87 ± 20/44) (*P* = 0.00)	(20/80 ± 20/48) (*P* = 0.00)	(20/67 ± 20/42) (*P* = 0.00)
CDVA	(20/32 ± 20/33)	(20/29 ± 20/30) (*P* = 0.01)	(20/28 ± 20/31) (*P* = 0.00)	(20/26 ± 20/30) (*P* = 0.00)	(20/24 ± 20/28) (*P* = 0.00)
Refraction (D)					
Sphere	0.05 ± 3.03	0.64 ± 3.14 (*P* = 0.12)	0.44 ± 2.82 (*P* = 0.55)	0.50 ± 2.92 (*P* = 0.10)	0.21 ± 2.43 (*P* = 0.61)
SE	−1.97 ± 3.19	−0.98 ± 3.09 (*P* = 0.00)	−1.38 ± 2.58 (*P* = 0.03)	−1.33 ± 3.11 (*P* = 0.01)	−1.23 ± 2.46 (*P* = 0.05)
Cylinder	−4.03 ± 2.53	−3.27 ± 2.21 (*P* = 0.00)	−3.65 ± 2.62 (*P* = 0.01)	−3.66 ± 2.41 (*P* = 0.00)	−2.88 ± 2.00 (*P* = 0.00)
CCT (*μ*m)	451 ± 45	425 ± 58 (*P* = 0.00; *n* = 21)	436 ± 45 (*P* = 0.00; *n* = 31)	441 ± 58 (*P* = 0.81; *n* = 26)	460 ± 47 (*P* = 0.15; *n* = 50)

UDVA: uncorrected distance visual acuity; CDVA: corrected distance visual acuity; SE: spherical equivalent; CCT: central corneal thickness.

**Table 2:** Topographic changes during 1-year followup.

Parameter	Preoperative (*n* = 61)	12 m postoperatively (*n* = 61)
Precisio		
PE (*μ*m)	71.56 ± 31.31	66.48 ± 28.81 (*P* = 0.01)
IRI (*μ*m)	45.45 ± 22.60	42.18 ± 22.54 (*P* = 0.01)
Asym (D)	9.05 ± 5.50	8.12 ± 5.58 (*P* = 0.01)
OPD		
Mean *K* (D)	46.97 ± 5.21	46.77 ± 5.31 (*P* = 0.06)
Max *K* (D)	55.55 ± 6.01	54.98 ± 5.78 (*P* = 0.02)
DSI	10.54 ± 5.30	9.71 ± 5.01 (*P* = 0.00)
*S* 3, 5, 7	1.40 ± 0.80	1.32 ± 0.80 (*P* = 0.04)
*S* 4, 6, 8	0.33 ± 0.21	0.34 ± 0.33 (*P* = 0.81)
Total HOA	4.80 ± 2.93	4.54 ± 2.72 (*P* = 0.05)

PE: posterior elevation; IRI: irregularity index; Asym: asymmetry within 5 mm zone; DSI: differential sector index; HOA: higher order aberration.
